# Assessing the quality of the anaerobic environment — a method developed to support EUCAST disk diffusion of anaerobic bacteria

**DOI:** 10.1007/s10096-023-04622-9

**Published:** 2023-05-12

**Authors:** Ulrik Stenz Justesen, Jenny Åhman, Erika Matuschek, Gunnar Kahlmeter

**Affiliations:** 1 Department of Clinical Microbiology, Odense University Hospital, J. B. Winsløwsvej 21, 2, 5000 Odense, Denmark; 2 EUCAST Development Laboratory, Växjö, Sweden

**Keywords:** Anaerobic bacteria, Quality control, Anaerobic environment, Antimicrobial susceptibility testing, Disk diffusion

## Abstract

The purpose of this study is to establish a method for assessing the anaerobic environment for EUCAST disk diffusion antimicrobial susceptibility testing (AST) of anaerobic bacteria on fastidious anaerobe agar with 5% mechanically defibrinated horse blood (FAA-HB). The method utilizes the association between a decrease in the metronidazole disk zone diameter and increasing oxygen levels with an aerotolerant *Clostridium perfringens* strain DSM 25589 (CCUG 75076 and NCTC 14679). The *C. perfringens* strain was tested on FAA-HB with a McFarland 1 inoculum and a metronidazole 5 μg disk. FAA-HB was incubated for 16–20 h at 35–37°C. The association between oxygen levels (0, 0.16, 1, 2, and 4% oxygen) and the metronidazole zone diameter was determined. Reproducibility at 0% oxygen was investigated as part of a European multi-centre study of disk diffusion of anaerobic bacteria. The median zone diameters (*n*=12) at each oxygen level were 29 mm (0%), 21 mm (0.16%), 16 mm (1%), 15 mm (2%), and 15 mm (4%). The metronidazole zone diameters at 0% oxygen from the multi-centre reproducibility-study had a median of 29 mm and a 95%-percentile range of 25–33 mm (*n*=236). Only one reading was below 25 mm. Based on our results, a zone diameter of ≥25 mm using a metronidazole 5 μg disk and the *C. perfringens* strain, tested with EUCAST recommendations, can be used to indicate that the anaerobic environment is of sufficient quality for culture and disk diffusion AST. EUCAST has included the method as part of the quality control for AST of anaerobic bacteria.

**Supplementary Information:**

The online version contains supplementary material available at 10.1007/s10096-023-04622-9.

## Introduction

A strict anaerobic environment is essential for the culture and antimicrobial susceptibility testing (AST) of anaerobic bacteria. Even small amounts of oxygen (<0.1%) in the atmosphere can result in no-growth of anaerobic bacteria or misleading susceptibility testing results. Colour changes with methylene blue or resazurin (chemical indicators) can be used as a control of the anaerobic environment [1]. However, they can remain negative although low levels of oxygen are present and will only display the existing atmosphere and not changes over time. The growth of strict anaerobic bacteria, such as *Fusobacterium nucleatum* ATCC 25586, can be used as a biological indicator, but it can be difficult to assess the result. No growth can be a result of low levels of oxygen or that the strain was already nonviable at inoculation.

We have previously published a method for quality control (QC) of the anaerobic environment, which was developed in the 1980s [2]. The method utilizes an aerotolerant *Clostridium perfringens* strain DSM 25589 (CCUG 75076 and NCTC 14679) which can grow in the presence of oxygen (spore suspension tested on Brucella blood agar). Metronidazole is dependent on reduction in an anaerobic environment to be active, and the effect of oxygen can be directly observed by a decrease in the metronidazole 5 μg zone diameter with increasing oxygen levels.

EUCAST has developed a disk diffusion method for AST of anaerobic bacteria based on the fastidious anaerobe agar supplemented with 5% mechanically defibrinated horse blood (FAA-HB) and a McFarland 1 inoculum [3, 4]. We wanted to see if we could use the EUCAST method, with the *C. perfringens* strain to develop a method, which could be used for assessing the anaerobic environment.

The purpose of this study was first to validate the method on the new medium FAA-HB with a McFarland 1 inoculum and to investigate the association between the metronidazole zone diameter and oxygen levels for *C. perfringens* DSM 25589. The second purpose was to establish a zone diameter cut-off value indicating a sufficient anaerobic environment and to test reproducibility over time based on the new method.

## Materials and methods

The *C. perfringens* DSM 25589 strain was cultured from freezer storage (−80°C) on FAA-HB (SSI Diagnostica, Hillerød, Denmark) for 16–20 h at 35–37°C in an anaerobic jar (Anoxomat jar system, MART Microbiology, Drachten, the Netherlands) in an anaerobic atmosphere of 10% H_2_, 10% CO_2_, and 80% N_2_. Before further use, the strain was subcultured at least once.

### Association between the metronidazole zone diameter and oxygen level

Two FAA-HB plates were inoculated with a McFarland 1 suspension of the *C. perfringens* strain with a cotton swab using an automatic plate rotator and a metronidazole 5 μg disk (Oxoid/Thermo Fisher Scientific, Basingstoke, UK) was placed on the plates. Plates were incubated in atmospheres with the following levels of oxygen: 0% (10% H_2_, 10% CO_2_, and 80% N_2_), 0.16%, 1%, 2%, and 4% using the Anoxomat jar system on six different days (*n*=12 per oxygen level). The plates were incubated for 16–20 h at 35–37°C. Growth was confluent after 16–20 h. Zone diameters were measured at 100% inhibition. This part of the study was performed at the Department of Clinical Microbiology, Odense University Hospital, Denmark.

### Quality control

*F. nucleatum* ATCC 25586, which will not grow or grow poorly at 0.16% oxygen, was chosen as a biological growth indicator for the first part of the study and included in every jar. A resazurin indicator was also included as a chemical indicator (Oxoid, Basingstoke, UK) [1].

### Reproducibility over time

The *C. perfringens* strain was also tested repeatedly in an anaerobic atmosphere to establish reproducibility and a cut-off value, which would indicate a sufficient anaerobic environment. Testing was performed at the EUCAST Development Laboratory, Växjö, Sweden; the Department of Clinical Microbiology, Odense University Hospital; and at 16 other laboratories participating in a European multi-centre study to validate the disk diffusion method for anaerobic bacteria, in which the *C. perfringens* strain was included on each test day. Testing was performed with the available anaerobic atmosphere at the local departments, including gas-generating systems, like the Anoxomat, anaerobic workstations and jars with gas-generating envelopes. Testing was performed on several batches of FAA-HB and on both in-house prepared and commercial (SSI Diagnostica, Hillerød, Denmark) plates. At Odense university Hospital, the impact of using the cotton swab with and without removing excess fluid was also tested (two plates on 6 different days).

## Results

### Association between the metronidazole zone diameter and oxygen level

**Fig. 1:**
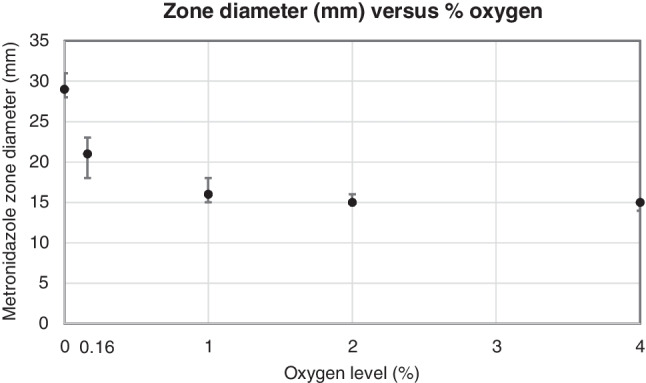
Results from the first part of the study. The metronidazole 5 μg zone diameter (mm) on FAA-HB versus the oxygen level (%) in the Anoxomat jar system. The medians with ranges are shown (*n*=12 at each oxygen level)

The zone diameter medians from the McFarland 1 suspension at each oxygen level were 29 mm (0%), 21 mm (0.16%), 16 mm (1%), 15 mm (2%), and 15 mm (4%), and the maximum range was 5 mm (Fig. 1). Inoculated FAA-HB plates with a metronidazole 5 μg disk, incubated at 0%, 0.16%, 1%, and 2% oxygen, are shown in Online Resource 1.

Throughout the first part of the study, growth of the biological indicator *F. nucleatum* ATCC 25586 was observed and the resazurin indicator turned white from pink on each test day at 0% oxygen. No growth of *F. nucleatum* ATCC 25586 was observed at 0.16% oxygen or above and the resazurin indicator remained pink.

### Reproducibility over time

**Fig. 2:**
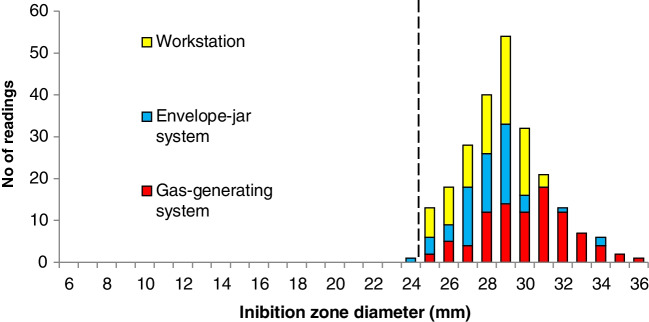
Reproducibility results from 18 laboratories and different anaerobic environments. Metronidazole 5 μg inhibition zone diameters on FAA-HB and *Clostridium perfringens* DSM 25589 (*n*=236)

Figure 2 demonstrates reproducibility of zone diameter readings (*n*=236) over time when tested at the EUCAST Development Laboratory, Växjö, Sweden; the Department of Clinical Microbiology, Odense University Hospital; and at 16 other laboratories participating in the European multi-centre study. The range was 24–36 mm with a median of 29 mm. The 95% percentile range and the interquartile range were 25–33 mm and 28–30 mm, respectively. Only one of 236 readings was below 25 mm. The median zone diameters from the gas-generating systems were 30 mm (*n*=93), anaerobic workstations 29 mm (*n*=80), and envelope-jar based systems 28 mm (*n*=63) (Fig. 2). There was no impact on zone diameters depending on the removal of excess fluid or not.

## Discussion

The previously published method developed using a spore suspension on BBA worked well with the FAA-HB and a McFarland 1 inoculum. A significant decrease in the metronidazole zone diameter from 29 to 21 mm was already observed from 0 to 0.16% oxygen. This makes the method very sensitive to even small amounts of oxygen, as a strict anaerobic environment is necessary to obtain confluent growth with the *C. perfringens* strain and a metronidazole zone diameter above the cut-off value. The reproducibility data from the many laboratories participating in the multi-centre trial established the robustness of the method as only minor variation was seen. The results also demonstrated that there was very little difference between the different methods for achieving an anaerobic environment.

The *C. perfringens* DSM 25589 strain is available from three culture collections (DSMZ, CCUG, and NCTC). EUCAST has included the method as part of the QC for AST of anaerobic bacteria, and the method is very easy to adapt in any laboratory performing anaerobic bacteriology [4].

Based on our results with the metronidazole 5 μg disk and the *C. perfringens* DSM 25589 tested with the EUCAST recommendations for AST of anaerobic bacteria with disk diffusion, an inhibition zone diameter of ≥25 mm can be used to indicate that the anaerobic environment is of sufficient quality for culture and disk diffusion AST. This is of particular importance for correct interpretation of metronidazole susceptibility test results.

## Supplementary information

ESM 1 (PDF 346 kb)
