# Supplementary material for: Assessing the quality of the anaerobic environment — a method developed to support EUCAST disk diffusion of anaerobic bacteria
Source: Eur J Clin Microbiol Infect Dis. 2023 May 12;42(7):895–8. doi: 10.1007/s10096-023-04622-9 (PMC10267253; doi:10.1007/s10096-023-04622-9)

ORIGINAL ARTICLE

Assessing the quality of the anaerobic environment – a method developed to support EUCAST disk diffusion of anaerobic bacteria

Ulrik Stenz Justesen[1,*], Jenny Åhman[2], Erika Matuschek[2], Gunnar Kahlmeter[2]

[1] Department of Clinical Microbiology, Odense University Hospital, Odense, Denmark

[2] EUCAST Development Laboratory, Växjö, Sweden

*Corresponding author: Ulrik Stenz Justesen, Department of Clinical Microbiology, Odense University Hospital, J. B. Winsløwsvej 21, 2., 5000 Odense C, Denmark. E-mail: ulrik.stenz.justesen@rsyd.dk Telephone: +45 6541 5749. ORDCID: 0000-0002-6130-1902

Supplementary Information

Online Resource 1 Results from the first part of the study. *Clostridium perfringens* DSM 25589 McFarland 1 suspension with a metronidazole 5 µg disk. Top left: 0%, top right: 0.16%, bottom left: 1% and bottom right 2% oxygen. The zone diameters are decreasing with increasing levels of oxygen

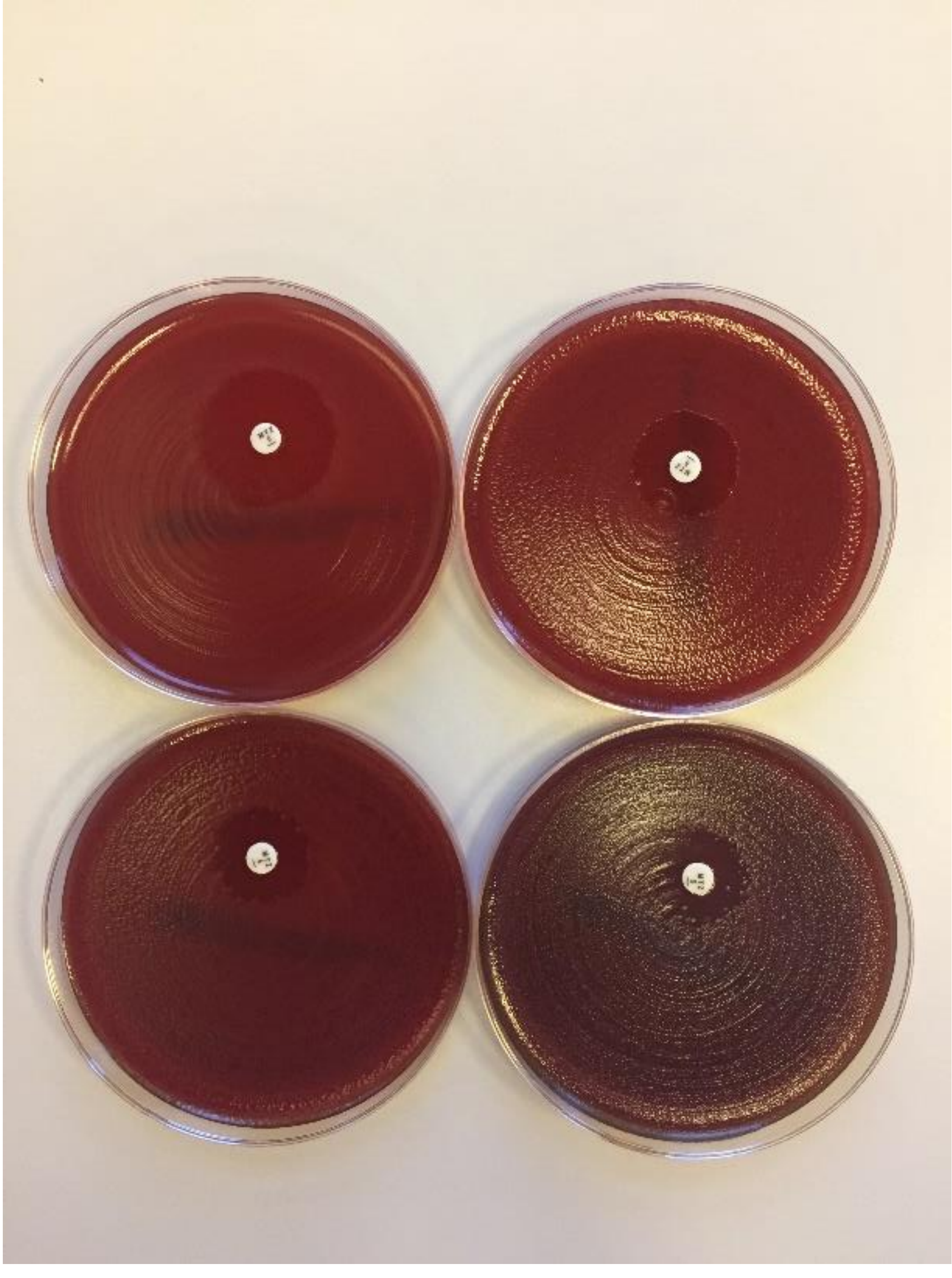

Supplement: Supplementary file 1 — (PDF 346 kb) [file 10096_2023_4622_MOESM1_ESM.pdf]
